# Action of Belladonna in Disease of the Cornea

**Published:** 1867-12

**Authors:** Joseph S. Hildreth

**Affiliations:** Chicago, Ill.


### ACTION OF BELLADONNA IN DISEASE OF THE CORNEA.

By JOS. S. HILDRETH, M.D., Chicago, Ill.

The use of belladonna, or other mydriatic, in some forms of corneal disease, is indispensable; while in others it becomes unnecessary, and often injurious.

From the complex structure of the eye, rendering it susceptible to numerous pathological changes, arises this apparently diverse action.

In the normal eye, the cornea is endowed with a delicate sensitiveness to the lightest touch; the pupil quickly responds to the influence of atropia, and its effects are quite persistent. This corneal sensitiveness remains, whether the pupil be dilated or contracted.

But clinical observation has demonstrated a condition of the eye in which the nervous integrity of the cornea is so disturbed as to cause a peculiar state of anæsthesia; the dilatability of the pupil is correspondingly lessened, and the effects of atropia are of shorter duration.

By gently touching the cornea with the point of a small camel's hair pencil, previously wet and stripped quite dry, or the point of a small roll of soft paper slightly moistened, the degree of anæsthesia is determined.

By instilling one drop of a solutiou of atropia of definite strength* within the lids at intervals of fifteen minutes, the dilatability of the pupil can be estimated.

These signs are pathognomonic of a condition which appears to be due to some defect of nervous action, which causes contraction and congestion of the ciliary ring.

The symptoms may be acute, with more or less congestion, or chronic without it.

There is another distinct form of corneal anæsthesia, which occurs in all glaucomatous affections. It is invariably produced by intra-ocular tension, and with a pupil dilated or readily dilatable; and, therefore, materially differs from that above described.

As there is no intra-ocular tension, and dilatability of the pupil is invariably diminished in the former, I have designated it as anæsthesia of the cornea and radiating fibres of the iris *without intra-ocular tension*, in contradistinction to the latter.

It is found that, as the depressed vitality of the cornea improves, normal dilatability of the pupil returns. To dilate and so maintain the pupil, relieves the anæsthetized cornea.

Nov. 30, 1866. Mr. B——, aged 35, bookkeeper. Slightly scrofulous and debilitated by overwork.

Four months previous had an attack of iritis in left eye which lasted two weeks. It was uncomplicated; no injury resulted; accommodation and vision were good. Was able to use both eyes without difficulty on the 28th. Felt trouble in the left eye the 29th, for which no cause can be assigned.

An irregular transparent ulceration quite deep, three millimetres wide, extends from lower margin of cornea four millimetres towards the pupil. The adjoining parts appear slightly infiltrated. The cornea is anæsthetized, especially the inferior

* Four grains of neutral sulphate of atropia to one ounce of water.

three-fourths. The pupil, smaller than the opposite, feebly responds to reflex movements and light. No other apparent disturbance of cornea or iris. Slight perikeratic injection. No inflammation or anæsthesia of conjunctiva, sclerotic, or lids. No evidence of paralysis of any part. Moderate photophobia and pain in central parts of globe.

Three drops of atropia solution, instilled within the lids five times during one hour and a-half, produce dilation of the pupil, and diminish corneal anæsthesia.

To apply two drops of same solution, within the lids, four times during the next twenty-four hours, and keep the eye well shaded. No other treatment.

Dec. 1st. Pupil more dilated; corneal anæsthesia nearly removed.

The ulceration is covered by a grayish exudation, and the remainder of the cornea is transparent. Perikeratic injection has nearly disappeared, and pain abated. Continue treatment.

Dec. 2d. Pupil well dilated; cornea quite free from anæsthesia; reparative exudation augmented. Continue treatment.

Dec. 4th. Pupil largely dilated, and no anæsthesia of cornea. Ulceration improving; no perikeratic injection or pain. To apply one drop of atropia solution twice daily.

The tongue being coated, with sensation of dryness of the fauces, muriate of ammonia mixture ordered.*

Dec. 10th. Repair of cornea quite complete. A few delicate vessels pass from its border, a short distance upon the newly formed material. Cornea free from anæsthesia, and pupil largely dilated.

Muriate of ammonia mixture, and atropia discontinued. Brown's citrine ointment to be applied three times weekly to the conjunctiva. To take a tonic mixture of bark and iodine.

Dec. 28th. The ulceration has healed, leaving a slight bluish trace. The pupil free in its movements, responds to reflex movements and light. Cornea free from anæsthesia. Vision and accommodation good.

In this case congestion and contraction of the ciliary ring produced anæsthesia of the cornea and radiating fibres of the iris. The patient being scrofulous and debilitated, keratic ulceration speedily followed.

* R.—Ammoniæ Muriatiss, ---------- 1 dram;
Potassæ Chloratis, ---------- ss dram;
Aquæ Destillatæ, ---------- 3 f oz.
Syrupi Auranti, ---------- 1 f oz.—M.

Sig.—One teaspoonful from three to six times daily.

Atropia, by relieving the congestion and contraction of the ciliary ring, restored the nervous integrity of the cornea, and allowed the reparative process to go on.

Sept., 1866. W——, aged 18, printer. Constitution good, little debilitated. Cornea largely nebulous; the epithelium and parts immediately beneath being disturbed. Pupil partially dilates with free use of atropia.

With contracted pupil, cornea anæsthetized; with dilated pupil, nearly free from anæsthesia. No other apparent disturbance in cornea or iris. No perikeratic injection, or inflammation of sclerotic, conjunctiva, or lids. No evidence of paralysis of any part, or anæsthesia of conjunctiva, lids, or face. Pain in central parts of globe.

This patient was under observation for over six months. Shortly after commencement, he took a small quantity of iodide of potassium. No other general treatment was employed.

The pupil was dilated by atropia, and red precipitate ointment* applied within the lids once to three times weekly. Towards the close, the yellow amorphous oxide of mercury, in ointment,† was substituted four times, at intervals of a week.

While the pupil was kept fully under the influence of atropia, progress on the part of the cornea, was very manifest; but as soon as the pupil was allowed to contract, the cornea made no improvement.

This was frequently repeated, and, as in all similar cases, with the same result.

April 13, 1867. Pupil normally dilatable; reflx movements, vision, and accommodation are good.

In this case, contraction of the ciliary ring, without apparent congestion, produced corneal anæsthesia and diminished dilatability of the pupil.

The condition of the cornea indicated stimulation; but this could not take effect until the ciliary ring had been relaxed by belladonna, and the corneal nerves relieved.

* R.—Hydg. Oxyd. Rub., ---------- 8 grains;

Hydg. Chld. Mite, ---------- 12 grains;

Pulv. Camph., ---------- } aa 1 grain;

Zinci Sulph., ---------- }

Axungiæ, ---------- 2 drams;

Misce bene. Sig.—A piece the size of a grain of wheat within the lids.

† R.—Hydg. Oxyd. flavi, ---------- ss dram;
(via humida parati)
Axungiæ, ---------- ss oz.
Misce bene.
Sig.—To be used in the same manner as the red precipitate ointment.

From numerous observations, the above cases have been selected as representing acute and chronic forms of anæsthesia of the cornea, and diminished dilatability of the pupil arising from those disturbances in the ciliary ring specially described, and illustrate the action of belladonna in this class of cases.

Injurious results have not been observed to follow its protracted use. But, when these conditions are absent, mydriatics are not requisite in uncomplicated keratic disease, and often become injurious, by producing atropinism of the eye.

Hence, in affections of the cornea, occurring: *a.* With anæsthesia and diminished dilatability of the pupil, belladonna is indicated. *b.* With normal dilatability of the pupil and absence of corneal anæsthesia, belladonna is not required.

The conditions indicating the use of mydriatics frequently exist to a degree requiring surgical interference to enable the drug to take effect.

Division of the ciliary ring is then often required.

Inasmuch as more or less of the aqueous humor escapes during this operation, the beneficial results are attributed, by some, to diminished intra-ocular tension. By direct experiment it has been demonstrated that relief of corneal anæsthesia and diminished dilatability of the pupil, in these cases, are the result of a thorough division of the ciliary ring, and not due to evacuation of the aqueous humor.

Paracentesis of the anterior chamber may in some cases of this class prove serviceable by diminishing congestion of the ciliary ring, but not otherwise.

In one instance, contraction of the ciliary ring was found to co-exist with glaucoma.

Iridectomy necessarily removed all intra-ocular tension, but failed to relieve the corneal anæsthesia. Division of the ciliary ring fifteen minutes afterwards at once accomplished that result.

The length and title of this paper preclude anything further upon surgical treatment.—*Trans. of Amer. Med. Association.*

